# The Arena for February

**Published:** 1897-02

**Authors:** 


### BOOK NOTICES.

THE ARENA for February, 1897, will have the following table of contents:

"The New Education," Hon. W. T. Harris, LL. D., U. S. Commissioner of Education; "Art for Truth's Sake in the Drama," James A. Herne; "The Civic Church," Paul Tyner; "Recompense:" a Poem, Charles G. Miller; "Our Arid Lands," Judge J. S. Emery, National Irrigation Commissioner; "Emerson's 'Sphinx,'" Charles Malloy; "The Telegraph Monopoly:" Part XII, Prof. Frank Parsons; "Giosue Carducci," Mary Sifton Pepper; "Pneumatology, Science of Spirit," Lucy S. Crandall; "The Problem of the Novel," Annie Nathan Meyer; "Should Hawaii be Annexed?" John R. Musick; "William Morris:" a Sonnet, O. E. Olin; "The Effects of Nicotine," Prof. Jay W. Seaver, A. M., M. D., Yale University; "The National Council of Women," Mary Lowe Dickinson, President National Council of Women; "A Court of Medicine and Surgery;" a Symposium, Henry O. Marcy, A. M., M. D., LL. D.; Hon. Elroy M. Avery, Ph. D., LL. D.; Edward M. Grout; Thaddeus B. Wakeman; Landon Carter Gray, M. D., and others; "On the Threshold: A Psychic Experience," Genevieve Thorndike Clark; Book Reviews: "Life Work of Thomas L. Nugent," "An American Idyll;" "The Duke and the Humanitarian;" "Deborah, the Advanced Woman;" "Life's Gateways."

The Arena Publishing Company, Copley Square, Boston, Mass. $3.00 a year. Single copies, 25 cents.

